# Bleeding and Thrombotic Risk in Cirrhosis: Hemostatic Profiles from a Single-Center Experience

**DOI:** 10.3390/life16061003

**Published:** 2026-06-15

**Authors:** Teodor Cabel, Oana-Mihaela Plotogea, Madalina Ilie, Vasile Sandru, Christopher Pavel, Raluca-Ioana Dascalu, Gabriel Constantinescu, Simona-Olimpia Dima, Mariana Mihaila

**Affiliations:** 1 Faculty of Medicine, “Carol Davila” University of Medicine and Pharmacy, 050474 Bucharest, Romania; teodor.cabel@drd.umfcd.ro (T.C.); drsandruvasile@gmail.com (V.S.); christopher.pavel@gmail.com (C.P.); raluca-ioana.dascalu@rez.umfcd.ro (R.-I.D.); gabrielconstantinescu63@gmail.com (G.C.); dima.simona@gmail.com (S.-O.D.); 2 Department of Gastroenterology, Clinical Emergency Hospital of Bucharest, 014461 Bucharest, Romania; 3 Department of Gastroenterology, Emergency Clinical Hospital Prof. Dr. Agrippa Ionescu, 011356 Bucharest, Romania; 4 Department of General Surgery and Liver Transplantation, Fundeni Clinical Institute, 022328 Bucharest, Romania; 5 Department of Internal Medicine, Fundeni Clinical Institute, 022328 Bucharest, Romania; m.mihaila14@gmail.com

**Keywords:** chronic liver disease (CLD), hemostatic imbalance (HI), gastrointestinal bleeding (GIB), portal vein thrombosis (PVT), anticoagulation therapy (AT)

## Abstract

Background/Objectives: Cirrhosis is characterized by a fragile and unstable hemostatic balance, predisposing patients to both hemorrhagic and thrombotic complications, particularly in the context of chronic liver disease (CLD). This dual risk presents significant challenges for clinical management and complicates therapeutic decisions. The objective of this study was to evaluate coagulation profiles in cirrhotic patients and their association with bleeding and thrombotic events, with the aim of identifying predictors of hemostatic complications. Methods: A retrospective study was conducted on patients with cirrhosis admitted to a tertiary liver transplant center between February 2022 and March 2024. Inclusion criteria required the availability of extended coagulation data in medical records. Demographic, clinical, and laboratory parameters were collected and analyzed to assess associations with thrombotic events and upper gastrointestinal bleeding (UGIB). Results: Fifty-nine patients met the inclusion criteria (mean age 53.4 ± 10.3 years), of whom 62.7% were male. UGIB occurred in 18.6% of patients, thrombotic events in 20.3%, and portal vein thrombosis in 16.9%. Routine coagulation parameters, including international normalized ratio (INR) and activated partial thromboplastin time (APTT), did not demonstrate significant predictive value for either bleeding or thrombotic events. Conclusions: Patients with cirrhosis are simultaneously at risk for both bleeding and thrombosis, reflecting the fragile and complex nature of hemostatic regulation in advanced liver disease (ALD). Standard laboratory coagulation tests are inadequate for accurate risk stratification in this setting. These findings highlight the limitations of conventional coagulation tests and support the need for further studies evaluating advanced hemostatic assessment tools in patients with cirrhosis.

## 1. Introduction

Cirrhosis represents the final stage of chronic liver disease and remains a major cause of morbidity and mortality worldwide [1]. Regardless of etiology, progressive hepatic fibrosis leads to portal hypertension, liver dysfunction, and a wide spectrum of complications that frequently require hospitalization [2,3,4]. Patients with advanced cirrhosis often experience episodes of acute decompensation, including gastrointestinal bleeding, infections, hepatic encephalopathy, hepatorenal syndrome, and thrombotic complications, all of which contribute to poor clinical outcomes [5,6,7].

Among the complications of cirrhosis, gastrointestinal bleeding and bacterial infections are the most frequent causes of acute decompensation. However, thrombotic events, particularly portal vein thrombosis, have gained increasing attention because of their impact on portal hypertension, disease progression, and overall prognosis [2,3,4,5,6]. These observations highlight the complex hemostatic disturbances that characterize advanced liver disease and contribute substantially to morbidity and hospitalization.

Hemostatic imbalance is a hallmark of cirrhosis and predisposes patients to both bleeding and thrombotic complications, creating a clinical paradox that complicates risk assessment and therapeutic decision-making [8]. Patients with cirrhosis are particularly vulnerable to upper gastrointestinal bleeding, mainly as a consequence of portal hypertension and gastroesophageal varices. Previous studies have reported that 25–35% of cirrhotic patients experience variceal bleeding during the course of the disease, with substantial short-term mortality despite advances in treatment [9,10].

At the same time, cirrhotic patients paradoxically exhibit an increased risk of thrombosis, particularly within the portal venous system. Portal vein thrombosis occurs in approximately 10–20% of patients with cirrhosis and may further aggravate portal hypertension and liver dysfunction [11]. These observations challenge the traditional concept of cirrhosis as a purely hemorrhagic disorder and support the current understanding of cirrhosis as a state of fragile and rebalanced hemostasis [12].

Conventional methods of assessing bleeding or thrombotic risk, such as prothrombin time (PT) and platelet count, are often inadequate in patients with cirrhosis. Although routinely used, these values do not reflect the proper balance between pro- and anticoagulant forces in liver disease and may miss early signs of decompensation.

It is important to note that conventional coagulation parameters, such as platelet count and INR, are poor predictors of bleeding risk in this population. This is largely due to compensatory changes, including increased von Willebrand factor (VWF) levels, reduced ADAMTS13 activity, and relatively preserved thrombin generation [13,14]. Consequently, bleeding events are more frequently driven by portal hypertension and structural lesions (e.g., varices, mucosal ulcers) than by a true coagulopathy [15].

The early identification of hemostatic disturbances in patients with liver disease requires advanced diagnostic modalities, such as thromboelastography (TEG) and rotational thromboelastometry (ROTEM) [16,17]. These viscoelastic assays provide a dynamic evaluation of the coagulation process by assessing the viscoelastic properties of whole blood in real-time, including clot formation, stabilization, and fibrinolysis [18,19]. In contrast to conventional coagulation tests, which evaluate isolated components of the hemostatic system, TEG and ROTEM offer a comprehensive assessment that more closely approximates the in vivo coagulation environment.

These technologies enable the simultaneous analysis of the complex interactions among procoagulant and anticoagulant factors, fibrinolytic pathways, and cellular elements such as erythrocytes and leukocytes, throughout the phases of primary and secondary hemostasis as well as fibrinolysis [18,20,21]. Their clinical relevance is particularly evident in the management of bleeding, where they facilitate individualized transfusion strategies. TEG and ROTEM have been shown to reduce the incidence of unnecessary erythrocyte transfusions, enable earlier initiation of plasma therapy, and contribute to the prediction and prevention of future hemorrhagic episodes [19,20,22]. Unfortunately, these laborious investigations are not available in every hospital unit but only in tertiary centers.

The hemostatic imbalance in these patients stems from several pathophysiological factors. Studies show that up to 70% of patients with cirrhosis have thrombocytopenia (platelet count < 150,000/μL), mainly due to hypersplenism and the reduced production of thrombopoietin by the liver [23]. In addition, increased platelet adhesion to von Willebrand factor further alters the balance [15]. The hepatic synthesis of both procoagulant (fibrinogen, factor V) and anticoagulant (protein C, antithrombin) factors is impaired, creating a delicate balance that can tip either toward bleeding or clotting, depending on multiple external factors, such as infections of various origins, dehydration, or surgery [24].

Another key factor is the increased levels of factor VIII, which increase procoagulant activity in cirrhosis. There is evidence that factor VIII levels are increased by 50–100% compared with healthy controls, making these patients prone to thrombosis, particularly in the portal venous system [25]. Hyperfibrinolysis also contributes to both bleeding and coagulation risks, as clot breakdown may occur too rapidly or insufficiently [26]. Anticoagulant therapy in patients with cirrhosis has historically been approached with caution due to concerns about the pre-existing bleeding risks of this condition. However, recent studies have highlighted its importance in managing thrombotic complications without substantially increasing bleeding episodes. In particular, anticoagulation has been associated with reduced rates of hepatic decompensation and improved survival outcomes in patients with cirrhosis [27]. Both low molecular weight heparins (LMWHs) and vitamin K antagonists (VKAs) have been used effectively in this setting. Emerging evidence also suggests that direct oral anticoagulants may be safe for use in patients with stable cirrhosis, although further research is needed to confirm their efficacy and safety profiles [28].

Despite substantial advances in the understanding of rebalanced hemostasis, identifying cirrhotic patients at risk for bleeding or thrombosis remains challenging in routine clinical practice. Conventional coagulation tests frequently fail to reflect the complexity of hemostatic alterations, and the clinical significance of extended coagulation parameters remains incompletely understood.

Therefore, this study aimed to characterize the coagulation profile of cirrhotic patients who underwent extended coagulation testing and to investigate the relationship between coagulation abnormalities, upper gastrointestinal bleeding, and thrombotic events. We hypothesized that extended coagulation markers and natural anticoagulant factors may provide additional information beyond conventional coagulation tests and may help improve the assessment of hemostatic risk in patients with cirrhosis.

## 2. Materials and Methods

We conducted a retrospective observational study including adult patients with established liver cirrhosis admitted to a tertiary liver transplant center between February 2022 and March 2024. The diagnosis of cirrhosis was established based on clinical, laboratory, imaging, and/or histological findings documented in the medical records.

During the study period, 63 patients were screened for eligibility. Inclusion criteria were: age ≥ 18 years, confirmed diagnosis of liver cirrhosis, admission during the study period, and availability of a complete extended coagulation panel. Exclusion criteria were: active malignancy, absence of chronic liver disease, pregnancy, and incomplete clinical or laboratory data, including missing extended coagulation testing.

Four patients were excluded from the analysis: three because extended coagulation testing was not available and one because of active hepatocellular carcinoma. The final study cohort consisted of 59 patients.

Extended coagulation testing was not routinely performed in all patients with cirrhosis admitted during the study period. Because the primary objective of this study was to evaluate the relationship between extended coagulation parameters and bleeding or thrombotic events, only patients for whom a complete extended coagulation panel was available were eligible for inclusion. We acknowledge that this selection criterion may have introduced selection bias and may limit the generalizability of the findings.

All data were included in a database created solely for this article and statistically analyzed. For statistical analysis, we used the IBM SPSS v.26 software package and considered a *p* value below 0.05 statistically significant. Descriptive statistical results were expressed as means ± standard deviations and ranges or as medians and ranges for continuous variables. In addition, categorical variables were expressed as frequencies/absolute numbers with percentages. To test statistical hypothesis, we performed several tests according to our databases: the chi-square test and ANOVA unifactorial test.

The institutional review board waived informed consent due to the study’s retrospective nature. Patient data were anonymized to ensure confidentiality.

## 3. Results

Fifty-nine patients with cirrhosis who were admitted to our tertiary liver transplant center and underwent extensive coagulation testing were included in this study. The mean age of the cohort was 53.41 ± 10.29 years (range: 30–74 years; median: 53). Of the study population, 62.7% were male (*n* = 37) and 37.3% were female (*n* = 22). The predominant etiology of cirrhosis was alcohol-related liver disease, accounting for 71.2% of cases (*n* = 42), followed by hepatitis B infection (18.6%, *n* = 11), hepatitis D infection (11.9%, *n* = 7), and hepatitis C infection (6.8%, *n* = 4). Other etiologies included non-alcoholic fatty liver disease (NAFLD), autoimmune liver diseases, drug-induced cirrhosis, and miscellaneous causes. The patient population and their characteristics are presented in Table 1.

According to the Child–Pugh classification, most patients had advanced liver disease: 64.4% (*n* = 38) were classified as Child–Pugh class C and 33.9% (*n* = 20) as class B, while only 1 patient (1.7%) was classified as class A. Therefore, 98.3% of the study cohort consisted of patients with decompensated or advanced cirrhosis. This distribution is clinically relevant, as advanced liver dysfunction is associated with more pronounced alterations in hemostatic balance, portal hypertension, and an increased risk of both hemorrhagic and thrombotic complications. The mean MELD-Na score was 25.42 ± 7.28 (range: 14–42; median: 26). Liver transplantation was performed in 3.4% (*n* = 2) of the patients, while the remaining 96.6% (*n* = 57) did not undergo transplantation. Transjugular intrahepatic portosystemic shunt (TIPS) was performed in only one patient in each group, selected based on clinical indications and eligibility criteria. Decompensated cirrhosis was present in 79.7% of the patients (*n* = 47). Upper gastrointestinal bleeding (UGIB) occurred in 18.6% of patients (*n* = 11), while 81.4% (*n* = 48) did not have UGIB. The prevalence of thrombosis was 20.3% (*n* = 12), and portal vein thrombosis was observed in 16.9% of patients (n = 10), with no statistically significant difference between the UGIB group (9.1%, *n* = 1) and the non-UGIB group (18.8%, *n* = 9; *p* = 0.670). Thrombotic events other than portal vein thrombosis were observed in 6.8% of patients (*n* = 4), with no events in the UGIB group (*p* = 0.428).

Coagulation parameters were assessed in both groups (with and without UGIB). Table 2 shows the coagulation profiles of patients with and without GIB, highlighting the differences in laboratory parameters and the prevalence of thrombosis and anticoagulant use.

D-dimer levels were significantly lower in patients with UGIB (2317.18 ± 1988.97 ng/mL) than in those without UGIB (4171.10 ± 4370.32 ng/mL; *p* = 0.041). Factor VIII levels were significantly higher in the UGIB group (36.49 ± 26.25%) compared with the group without UGIB (21.40 ± 15.47%; *p* = 0.014). Other coagulation parameters, including INR, APTT, factors II, V, X, antithrombin III, protein C, and protein S, did not show significant differences between the two groups.

The association between coagulation parameters, natural anticoagulant factors, and the presence of varices or thrombosis was further examined. No statistically significant differences were observed, with the exception of INR, which was significantly lower in patients with varices (*p* = 0.025). Therefore, the lower INR in the varices group should not necessarily be interpreted as a reduced bleeding risk, but rather as another indicator of the complex and often misleading nature of conventional coagulation parameters in advanced liver disease. Table 3 and Table 4 present the extended coagulation profiles for the two patient groups analyzed.

The complications encountered were hepatic encephalopathy in 23.7% of patients (n = 14), spontaneous bacterial peritonitis in 13.6% (*n* = 8), and UGIB in 18.6% (*n* = 11). The mortality rate in this study group was 37.3% (*n* = 22).

Patients with UGIB had a significantly higher incidence of anemia (100%, *n* = 11) than those without upper gastrointestinal hemorrhage (54.2%, *n* = 26; *p* = 0.004). Thrombocytopenia was observed in 72.7% (*n* = 8) of patients with UGIB and in 70.8% (*n* = 34) of patients without UGIB (*p* = 0.608), but without statistical significance. Elevated creatinine levels were observed in 45.5% (*n* = 5) of patients in the UGIB group and in 33.3% (*n* = 16) of patients in the non-UGIB group (*p* = 0.336), partially influencing therapeutic management decisions.

## 4. Discussion

This study emphasizes the complex hemostatic changes in cirrhotic patients, underscoring the simultaneous risks of bleeding and thrombosis, as well as the difficulty of diagnosis in routine clinical practice. This duality aligns with the concept of “rebalanced hemostasis” in cirrhosis, where concomitant decreases in procoagulant and anticoagulant factors create a fragile balance [29].

Upper gastrointestinal bleeding was observed in 18.6% of the study population. Patients with UGIB had significantly higher factor VIII levels; however, the clinical significance of this finding remains uncertain, particularly given the limited sample size and the relatively small number of bleeding events observed in our cohort.

Conversely, several studies have demonstrated a positive association between elevated FVIII levels and an increased risk of thrombotic events, most notably portal vein thrombosis, which is also linked to greater disease severity [30]. Moreover, portal hypertension has been correlated with elevated serum factor VIII concentrations, and increased levels have been observed in patients with deep vein thrombosis, even in the absence of advanced chronic liver disease [31]. These findings underscore the need for further research to more precisely define the thrombotic risk profile in cirrhotic patients and to advance our understanding of the mechanisms contributing to hemostatic imbalance in this population.

However, traditional coagulation parameters, such as INR and APTT, did not effectively predict bleeding risk, consistent with findings from previous studies that question the reliability of these values in cirrhotic populations [32]. Consequently, there is increasing emphasis on comprehensive hemostatic assessments, such as thromboelastography or rotational thromboelastometry, to more accurately assess bleeding risk in this population [33]. The disadvantages of these advanced investigations are their high cost and their inability to be performed in most hospitals.

The incidence of portal vein thrombosis (PVT) in our study was 16.9%, consistent with the existing literature, which reports PVT prevalence rates ranging from 10% to 20% in patients with cirrhosis [34]. The occurrence of PVT, regardless of UGIB status, suggests that bleeding episodes do not necessarily confer protection against thrombotic events. This finding emphasizes the complex interplay between bleeding and clotting tendencies in cirrhosis and highlights the importance of vigilant monitoring for thrombotic complications, even in patients presenting with bleeding episodes [35].

Anticoagulation in patients with cirrhosis has traditionally been approached with caution because of concerns about bleeding risks; however, recent studies indicate that it can be safely administered in some cases, particularly in individuals with PVT, without substantially increasing the likelihood of bleeding complications [36].

Recent research suggests that anticoagulant therapy plays a crucial role in reducing the rates of hepatic decompensation and improving the overall survival outcomes in patients with cirrhosis [37]. Beyond the management of PVT, anticoagulant therapy has demonstrated broader benefits in patients with cirrhosis. Studies indicate that it prevents PVT, decreases hepatic decompensation, and improves survival rates. A randomized controlled trial reported that enoxaparin effectively prevented PVT, reduced hepatic decompensation, and improved survival in patients with advanced cirrhosis [38].

Low molecular weight heparins and vitamin K antagonists have been used successfully in this setting. In addition, emerging data suggest that direct oral anticoagulants (DOACs) may be a viable option for patients with stable cirrhosis, although further investigation is needed to confirm their safety and efficacy [39]. In particular, studies have associated DOACs with a lower risk of all-cause mortality compared with untreated patients, without a significant increase in bleeding complications [40]. However, these observations are based on evidence from previously published studies, as the present study was not designed to evaluate the impact of anticoagulant therapy on survival, hepatic decompensation, or other clinical outcomes.

Current clinical guidelines recommend anticoagulant therapy for acute or subacute non-neoplastic PVT in patients with cirrhosis, highlighting its therapeutic potential in this patient population [41]. However, the optimal timing for initiating anticoagulation remains uncertain, highlighting the need for further research to establish appropriate initiation strategies.

Although further prospective data are needed, findings from previous studies suggest a potential therapeutic role for anticoagulation in selected patients with cirrhosis.

The retrospective design of this study and the relatively small sample size may have limited the statistical power of the analyses and may affect the generalizability of our findings. Furthermore, because only patients who underwent extended coagulation testing were included, selection bias cannot be excluded. In addition, the absence of advanced hemostatic assessments, such as TEG or ROTEM, limits our ability to fully characterize the hemostatic profile of our cohort. Future prospective studies incorporating these comprehensive assessments and larger patient populations are warranted to improve our understanding of hemostatic dynamics in patients with cirrhosis.

## 5. Conclusions

This study supports the concept of cirrhosis as a state of fragile and rebalanced hemostasis characterized by concurrent bleeding and thrombotic risk. In our cohort, conventional coagulation parameters, including INR and APTT, did not reliably predict bleeding or thrombotic events, highlighting the limitations of standard coagulation testing in patients with advanced liver disease.

The prevalence of portal vein thrombosis observed in this study further emphasizes the prothrombotic potential of cirrhosis despite the coexistence of hemorrhagic complications. Although previous studies have reported potential benefits of anticoagulant therapy in selected patients with cirrhosis, the present study was not designed to evaluate treatment outcomes; therefore, no conclusions regarding the efficacy of anticoagulation can be drawn from our data.

Given the retrospective design, the relatively small sample size, and the lack of advanced viscoelastic testing such as TEG or ROTEM, these findings should be interpreted with caution and considered exploratory. Larger prospective studies are needed to further clarify the relationship between coagulation abnormalities and clinical outcomes and to improve risk stratification in patients with cirrhosis.

## Figures and Tables

**Table 1:** Patient population and characteristics.

Variables	
Age, years	53.41 ± 10.29 (range: 30–74; median: 53)
Gender	
Female	22 (37.3%)
Male	37 (62.7%)
Etiology	
Hepatitis B	11 (18.6%)
Hepatitis C	4 (6.8%)
Hepatitis D	7 (11.9%)
Alcohol	42 (71.2%)
NAFLD	1 (1.7%)
Autoimmune	2 (3.4%)
Drug-induced	1 (1.7%)
Other causes	3 (5.1%)
ChildPugh	
A	1 (1.7%)
B	20 (33.9%)
C	38 (64.4%)
MELDNa	25.42 ± 7.28 (range: 14–42; median: 26)
Hepatic transplant	
Yes	2 (3.4%)
No	57 (96.6%)
Decompensation	
Yes	47 (79.7%)
No	12 (20.3%)
Complications	
HE	14 (23.7%)
SBP	8 (13.6%)
Upper GI bleeding	11 (18.6%)
Death	22 (37.3%)

**Table 2:** Coagulation parameters between patients with or without upper gastrointestinal bleeding.

Variables	With UGIB (*n* = 11)	Without UGIB (*n* = 48)	*p*
Varices	11 (100%)	42 (87.5%)	0.272
Esophageal	8 (72.7%)	37 (82.2%)	0.516
Gastric	0 (0%)	1 (2.1%)	0.814
Esogastric	3 (27.3%)	4 (8.3%)	0.112
Thrombosis (Yes/No)	1/10 (9.1/90.9%)	11/37 (22.9/77.1%)	0.431
Portal vein thrombosis (Yes/No)	1/10 (9.1/90.9%)	1/10 (9.1/90.9%)	0.670
Other thrombosis (Yes/No)	0/11 (0/100%)	4/44 (8.3/91.7%)	0.428
Anticoagulant treatment (Yes/No)	2/9 (18.2/81.8%)	21/27 (43.8/56.2%)	0.174
INR	1.95 ± 0.605	2.10 ± 0.644	0.469
APTT	38.23 ± 9.02	41.43 ± 9.88	0.314
D-dimers	2317.18 ± 1988.97	4171.10 ± 4370.32	**0.041**
II factor	42.80 ± 16.54	38.08 ± 17.79	0.413
V factor	47.26 ± 19.25	43.10 ± 21.56	0.536
VIII factor	36.49 ± 26.25	21.40 ± 15.47	**0.014**
X factor	40.87 ± 20.70	41.50 ± 19.21	0.923
Antithrombin III	42.64 ± 21.44	32.41 ± 17.11	0.094
Pc value %	44.82 ± 20.56	37.74 ± 16.53	0.228
Ps value %	50.55 ± 31.31	44.70 ± 31.50	0.581

**Table 3:** Coagulation parameters for patients with or without varices.

Varices			
	Yes	No	*p*
INR	2.01 ± 0.6	2.62 ± 0.71	**0.025**
APTT	40.05 ± 9.67	47.82 ± 7.88	0.063
D-dimers	3989.17 ± 4241.34	2379.33 ± 1949.92	0.365
Fibrinogen	244.87 ± 108.54	194.5 ± 88.27	0.279
II factor	38.86 ± 16.6	39.9 ± 26.38	0.892
V factor	44.31 ± 21.52	40.07 ± 17.51	0.644
VIII factor	25.06 ± 19.21	16.72 ± 11.04	0.303
X factor	41.16 ± 19.09	43.33 ± 23.06	0.797
Antithrombin III	35.15 ± 18.16	27 ± 18.92	0.304
Pc value %	39.67 ± 17.62	34 ± 15.77	0.454
Ps value %	45.18 ± 31.75	51.22 ± 28.83	0.659

**Table 4:** Coagulation parameters for patients with or without thrombosis.

Thrombosis			
	Yes	No	*p*
INR	2.12 ± 0.63	1.97 ± 0.65	0.439
APTT	40.49 ± 8.72	41.69 ± 12.16	0.673
D-dimers	3744.14 ± 4182.03	4026.35 ± 3955.82	0.813
Fibrinogen	222.5 ± 96.3	282.35 ± 122.88	0.051
II factor	39.33 ± 16.76	38.06 ± 19.81	0.803
V factor	43.63 ± 22.4	44.49 ± 17.91	0.889
VIII factor	25.29 ± 20.37	21.57 ± 13.71	0.493
X factor	39.72 ± 18.65	45.51 ± 20.88	0.301
Antithrombin III	33.95 ± 17.06	35.24 ± 21.41	0.808
Pc value %	39.12 ± 16.66	39 ± 19.79	0.982
Ps value %	45.11 ± 32.99	47.64 ± 27.17	0.786

## Data Availability

Data that support the findings of this study and materials are available from the first author upon reasonable request.

## Abbreviations

The following abbreviations are used in this manuscript:

ALD	Advanced liver disease
APTT	Activated partial thromboplastin time
CLD	Chronic liver disease
DOACs	Direct oral anticoagulants
INR	International normalized ratio
LMWHs	Low molecular weight heparins
NAFLD	Non-alcoholic fatty liver disease
PT	Prothrombin time
PVT	Portal vein thrombosis
ROTEM	Rotational thromboelastometry
TEG	Thromboelastography
TIPS	Transjugular intrahepatic portosystemic shunt
UGIB	Upper gastrointestinal bleeding
VKAs	Vitamin K antagonists
vWF	von Willebrand factor

## References

[1] Moon A.M., Singal A.G., Tapper E.B. (2020). Contemporary Epidemiology of Chronic Liver Disease and Cirrhosis. Clin. Gastroenterol. Hepatol..

[2] Sepanlou S.G., Safiri S., Bisignano C., Ikuta K.S., Merat S., Saberifiroozi M., Poustchi H., Tsoi D., Colombara D.V., Abdoli A. (2020). The global, regional, and national burden of cirrhosis by cause in 195 countries and territories, 1990–2017: A systematic analysis for the Global Burden of Disease Study 2017. Lancet Gastroenterol. Hepatol..

[3] Asrani S.K., Devarbhavi H., Eaton J., Kamath P.S. (2019). Burden of liver diseases in the world. J. Hepatol..

[4] Cheemerla S., Balakrishnan M. (2021). Global Epidemiology of Chronic Liver Disease. Clin. Liver Dis..

[5] Moreau R., Jalan R., Gines P., Pavesi M., Angeli P., Cordoba J., Durand F., Gustot T., Saliba F., Domenicali M. (2013). Acute-on-chronic liver failure is a distinct syndrome that develops in patients with acute decompensation of cirrhosis. Gastroenterology.

[6] Mansour D., McPherson S. (2018). Management of decompensated cirrhosis. Clin. Med..

[7] Mokdad A.A., Lopez A.D., Shahraz S., Lozano R., Mokdad A.H., Stanaway J., Murray C.J., Naghavi M. (2014). Liver cirrhosis mortality in 187 countries between 1980 and 2010: A systematic analysis. BMC Med..

[8] O’Leary J.G., Greenberg C.S., Patton H.M., Caldwell S.H.J.G. (2019). AGA clinical practice update: Coagulation in cirrhosis. AGA Clinical Practice Update: Coagulation in Cirrhosis. Gastroenterology.

[9] De Franchis R., Faculty B.V. (2015). Expanding consensus in portal hypertension: Report of the Baveno VI Consensus Workshop: Stratifying risk and individualizing care for portal hypertension. J. Hepatol..

[10] Garcia-Tsao G., Sanyal A.J., Grace N.D., Carey W., Practice Guidelines Committee of the American Association for the Study of Liver Diseases, Practice Parameters Committee of the American College of Gastroenterology (2007). Prevention and management of gastroesophageal varices and variceal hemorrhage in cirrhosis. Am. J. Gastroenterol..

[11] Amitrano L., Guardascione M.A., Brancaccio V., Margaglione M., Manguso F., Iannaccone L., Grandone E., Balzano A. (2004). Risk factors and clinical presentation of portal vein thrombosis in patients with liver cirrhosis. J. Hepatol..

[12] Tripodi A., Mannucci P.M. (2011). The coagulopathy of chronic liver disease. N. Engl. J. Med..

[13] Elhence A., Shalimar (2023). Von Willebrand Factor as a Biomarker for Liver Disease—An Update. J. Clin. Exp. Hepatol..

[14] Ferreira C.M., Rocha T.R.F., Souza E.O., Carrilho F.J., d’Amico E.A., Farias A.Q. (2021). Preservation of thrombin generation in cirrhosis despite abnormal results of international normalized ratio: Implications for invasive procedures. Blood Coagul. Fibrinolysis.

[15] La Mura V., Reverter J.C., Flores-Arroyo A., Raffa S., Reverter E., Seijo S., Abraldes J.G., Bosch J., García-Pagán J.C.J.G. (2011). Von Willebrand factor levels predict clinical outcome in patients with cirrhosis and portal hypertension. Gut.

[16] Blasi A., Calvo A., Prado V., Reverter E., Reverter J.C., Hernández-Tejero M., Aziz F., Amoros A., Cardenas A., Fernández J.J.H. (2018). Coagulation failure in patients with acute-on-chronic liver failure and decompensated cirrhosis: Beyond the international normalized ratio. Hepatology.

[17] Lisman T., Leebeek F.W., de Groot P.G. (2002). Haemostatic abnormalities in patients with liver disease. J. Hepatol..

[18] Whiting D., DiNardo J.A. (2014). TEG and ROTEM: Technology and clinical applications. Am. J. Hematol..

[19] Campello E., Zanetto A., Bulato C., Maggiolo S., Spiezia L., Russo F.P., Gavasso S., Mazzeo P., Tormene D., Burra P. (2021). Coagulopathy is not predictive of bleeding in patients with acute decompensation of cirrhosis and acute-on-chronic liver failure. Liver Int..

[20] Brill J.B., Brenner M., Duchesne J., Roberts D., Ferrada P., Horer T., Kauvar D., Khan M., Kirkpatrick A., Ordonez C. (2021). The Role of TEG and ROTEM in Damage Control Resuscitation. Shock.

[21] Katsaras G., Sokou R., Tsantes A.G., Piovani D., Bonovas S., Konstantinidi A., Ioakeimidis G., Parastatidou S., Gialamprinou D., Makrogianni A. (2021). The use of thromboelastography (TEG) and rotational thromboelastometry (ROTEM) in neonates: A systematic review. Eur. J. Pediatr..

[22] Janko N., Majeed A., Commins I., Gow P., Kemp W., Roberts S.K. (2024). Rotational thromboelastometry predicts future bleeding events in patients with cirrhosis. Scand. J. Gastroenterol..

[23] Mitchell O., Feldman D.M., Diakow M., Sigal S.H. (2016). The pathophysiology of thrombocytopenia in chronic liver disease. Hepat. Med..

[24] Northup P.G., Caldwell S.H. (2013). Coagulation in liver disease: A guide for the clinician. Clin. Gastroenterol. Hepatol..

[25] Tripodi A., Salerno F., Chantarangkul V., Clerici M., Cazzaniga M., Primignani M., Mannuccio Mannucci P.J.H. (2005). Evidence of normal thrombin generation in cirrhosis despite abnormal conventional coagulation tests. Hepatology.

[26] Leebeek F.W., Rijken D.C. (2015). The fibrinolytic status in liver diseases. Semin. Thromb. Hemost..

[27] Turco L., de Raucourt E., Valla D.C., Villa E. (2019). Anticoagulation in the cirrhotic patient. JHEP Rep..

[28] Protopapas A.A., Savopoulos C., Skoura L., Goulis I. (2023). Anticoagulation in Patients with Liver Cirrhosis: Friend or Foe?. Dig. Dis. Sci..

[29] Singh A.D., Mucha S.R., Lindenmeyer C.C. (2022). Cirrhotic coagulopathy: A rebalanced hemostasis. Clevel. Clin. J. Med..

[30] Kalambokis G.N., Oikonomou A., Christou L., Kolaitis N.I., Tsianos E.V., Christodoulou D., Baltayiannis G. (2016). von Willebrand factor and procoagulant imbalance predict outcome in patients with cirrhosis and thrombocytopenia. J. Hepatol..

[31] Jiang S., Ai Y., Fan X., Huang X., Wu L., Ni L., Li F., Chen S. (2023). Increased Factor VIII Activity Is Predictive of the Occurrence of Portal Vein Thrombosis in Cirrhosis. Thromb. Haemost..

[32] Beattie W., Magnusson M., Hardikar W., Monagle P., Ignjatovic V. (2017). Characterization of the coagulation profile in children with liver disease and extrahepatic portal vein obstruction or shunt. Pediatr. Hematol. Oncol..

[33] Harrison M.F. (2018). The Misunderstood Coagulopathy of Liver Disease: A Review for the Acute Setting. West. J. Emerg. Med..

[34] Kataria S., Juneja D., Singh O. (2023). Approach to thromboelastography-based transfusion in cirrhosis: An alternative perspective on coagulation disorders. World J. Gastroenterol..

[35] Prakash S., Bies J., Hassan M., Mares A., Didia S.C. (2023). Portal vein thrombosis in cirrhosis: A literature review. Front. Med..

[36] Rautou P.-E., Caldwell S.H., Villa E. (2023). Bleeding and Thrombotic Complications in Patients With Cirrhosis: A State-of-the-Art Appraisal. Clin. Gastroenterol. Hepatol..

[37] Zhang Z., Zhao Y., Li D., Guo M., Li H., Liu R., Cui X. (2023). Safety, efficacy and prognosis of anticoagulant therapy for portal vein thrombosis in cirrhosis: A retrospective cohort study. Thromb. J..

[38] Guerrero A., Campo L.D., Piscaglia F., Scheiner B., Han G., Violi F., Ferreira C.N., Tellez L., Reiberger T., Basili S. (2023). Anticoagulation improves survival in patients with cirrhosis and portal vein thrombosis: The IMPORTAL competing-risk meta-analysis. J. Hepatol..

[39] Niu C., Zhang J., Himal K., Zhu K., Zachary T., Verghese B., Jadhav N., Okolo P.I., Daglilar E., Kouides P. (2024). Impact of anticoagulation therapy on outcomes in patients with cirrhosis and portal vein thrombosis: A large-scale retrospective cohort study. Thromb. Res..

[40] Senzolo M., Garcia-Pagan J.C. (2023). A major research gap: The use of anticoagulants in cirrhosis. J. Hepatol..

[41] Chen X., Pan J., Zhu Y. (2024). Anticoagulation for cirrhosis with portal vein thrombosis: Still a road to be explored. J. Hepatol..

